# How to Tackle Key Challenges in the Promotion of Physical Activity among Older Adults (65+): The AEQUIPA Network Approach

**DOI:** 10.3390/ijerph14040379

**Published:** 2017-04-04

**Authors:** Sarah Forberger, Karin Bammann, Jürgen Bauer, Susanne Boll, Gabriele Bolte, Tilman Brand, Andreas Hein, Frauke Koppelin, Sonia Lippke, Jochen Meyer, Claudia R. Pischke, Claudia Voelcker-Rehage, Hajo Zeeb

**Affiliations:** 1 Leibniz Institute for Prevention Research and Epidemiology—BIPS, 28359 Bremen, Germany; brand@leibniz-bips.de (T.B.); pischke@leibniz-bips.de (C.R.P.); zeeb@leibniz-bips.de (H.Z.); 2 Working Group Epidemiology of Demographic Change, Institute for Public Health und Nursing Research (IPP), Faculty for Human and Health Sciences, University of Bremen, 28359 Bremen, Germany; bammann@uni-bremen.de; 3 Geriatrisches Zentrum, Universität Heidelberg, Agaplesion Bethanien-Krankenhaus, 69126 Heidelberg, Germany; juergen.bauer@bethanien-heidelberg.de; 4 Media Informatics and Multimedia Systems, Department of Computing Science, Carl von Ossietzky University of Oldenburg, 26121 Oldenburg, Germany; Susanne.Boll@informatik.uni-oldenburg.de; 5 Department of Social Epidemiology, Institute for Public Health and Nursing Research, University of Bremen, 28359 Bremen, Germany; gabriele.bolte@uni-bremen.de; 6 Research Focus Health Sciences Bremen, University of Bremen, 28359 Bremen, Germany; 7 Department of Health Services Research, School of Medicine and Health Sciences, Carl von Ossietzky University of Oldenburg, 26111 Oldenburg, Germany; andreas.hein@uni-oldenburg.de; 8 Section Technology and Health for Humans, Jade University of Applied Sciences Oldenburg, 26121 Oldenburg, Germany; frauke.koppelin@jade-hs.de; 9 Department of Psychology and Methods, Jacobs University Bremen, 28759 Bremen, Germany; s.lippke@jacobs-university.de; 10 OFFIS—Institute for Information Technology, 26121 Oldenburg, Germany; jochen.meyer@offis.de; 11 Institute of Human Movement Science and Health, Faculty of Behavioral and Social Sciences, Chemnitz University of Technology, 09107 Chemnitz, Germany; claudia.voelcker-rehage@hsw.tu-chemnitz.de

**Keywords:** ageing, ageing research, older adults, physical activity

## Abstract

The paper introduces the theoretical framework and methods/instruments used by the Physical Activity and Health Equity: Primary Prevention for Healthy Ageing (AEQUIPA) prevention research network as an interdisciplinary approach to tackle key challenges in the promotion of physical activity among older people (65+). Drawing on the social-ecological model, the AEQUIPA network developed an interdisciplinary methodological design including quantitative/qualitative studies and systematic reviews, while combining expertise from diverse fields: public health, psychology, urban planning, sports sciences, health technology and geriatrics. AEQUIPA tackles key challenges when promoting physical activity (PA) in older adults: tailoring of interventions, fostering community readiness and participation, strengthening intersectoral collaboration, using new technological devices and evaluating intervention generated inequalities. AEQUIPA aims to strengthen the evidence base for age-specific preventive PA interventions and to yield new insights into the explanatory power of individual and contextual factors. Currently, the empirical work is still underway. First experiences indicate that the network has achieved a strong regional linkage with communities, local stakeholders and individuals. However, involving inactive persons and individuals from minority groups remained challenging. A review of existing PA intervention studies among the elderly revealed the potential to assess equity effects. The results will add to the theoretical and methodological discussion on evidence-based age-specific PA interventions and will contribute to the discussion about European and national health targets.

## 1. Introduction

Healthy ageing is one of the great challenges of the 21st century. By 2030, approximately 25%–30% of the population in the European Union (EU 27) will be 65 years and older [1,2]. Physical activity (PA) plays a major role in healthy ageing and is important for physical and mental health, well-being, quality of life, development of personal resources, social contacts and maintenance of independent living [3]. Physical inactivity has been identified as the fourth leading risk factor for global mortality [4]. It has major implications for the prevalence of numerous non-communicable diseases such as cardiovascular diseases, diabetes and cancer as well as their associated risk factors such as elevated blood pressure and blood glucose levels and overweight [5].

The World Health Organization (WHO) recommends at least 150 min of moderate-intensity aerobic PA or at least 75 min of vigorous-intensity aerobic PA throughout the week for persons aged 65 and above to prevent non-communicable diseases [5]. Further, for this age group, flexibility and strength training is recommended for at least two times per week. This training increases mobility skills and decreases the risk of falling [6,7]. Moderate training, for example outdoor walking, improves cardio-metabolic markers [8], resting blood pressure [9] and postprandial blood glucose response [10]. Reductions in body mass index and blood pressure lower the risk of type II diabetes mellitus and, through a variety of mechanisms, cardiovascular mortality risk [11]. Additionally, positive effects of exercise on mental health and cognition have been reported [12]. Conversely, age-associated chronic diseases, such as osteoarthritis and osteoporosis, and falls may strongly impair an individual’s ability to be physically active and lead to an accelerated loss of muscle mass (sarcopenia) in older age [13,14,15]. However, health benefits of exercise are not only restricted to healthy persons. They are also evident in the secondary and tertiary prevention of chronic diseases [16]. Although the beneficial effects of PA are well established, PA levels in the population at large and in the elderly are below WHO recommendations. Data from the national representative German Health Interview and Examination Survey for Adults (DEGS) show that 83.2% of women and 80.7% of men aged 60–69 are physically active for less than 150 min per week [17]. Therefore, it is of importance to increase PA levels among people aged 65 and above. Older adults vary considerably in their abilities, needs and motivations for PA due to differences in their biographical experiences, social circumstances and health status [18,19,20]. While the functional status declines with age, there is large dispersion in the physical fitness and differences in the speed of decline in older adults [21,22]. In AQUIPA, we focus on PA as contributor to primary prevention among healthy community-dwelling older adults.

The paper provides the rationale and study design of the Physical Activity and Health Equity: Primary Prevention for Healthy Ageing (AEQUIPA, http://www.aequipa.de) prevention research network. AEQUIPA aims to increase PA among older adults by addressing key challenges of population-based PA promotion, namely: (1) tailoring PA interventions to individual conditions (e.g., on basis of the Health Action Process (HAPA) model) [23]; (2) fostering community readiness and participation; (3) strengthening intersectoral collaboration; (4) using technological devices and (5) evaluating intervention generated inequalities. With its approach, AEQUIPA combines various disciplines and experts to tackle key challenges in the promotion of PA among older adults from various angles so as to improve the knowledge about theory and methods development, tailoring of intervention, technic use, and the role of intervention generated inequality and environmental factors. After completion of the various subprojects at the end of 2017, the results gained will be translated into guidance for policy makers and practitioners on how to tackle physical inactivity in persons over 65 years as well as how to create healthy living environments.

### 1.1. Key Challenges Influencing the Initiation and Maintenance of PA

According to Sallis and colleagues’ socio-ecological model [24], factors and conditions at multiple levels influence PA behaviour as individual characteristics (e.g., cognition, objective and subjective health, health-related behaviour, self-perception, planning, intention, PA level) and contextual factors (environment, setting, living and social condition) significantly affect and modify ageing processes in later life. Further, the use of new technologies can have a positive influence on PA levels. This is however affected by individual characteristics such as openness and technical affinity, and also factors such as socio-economic status and access to information and resources. Although PA intervention can also increase or reduce inequality, these aspects are rarely considered during the intervention development and implementation process. The AEQUIPA framework provides a combined approach to these aspects and aims for transdisciplinary and integrative research guided by the socio-ecological model.

#### 1.1.1. Tailoring PA Interventions to Individual Conditions

PA interventions seem to be effective if they target the essential factors relevant to the self-regulation of individuals [25]. Positive health effects of PA are already evident after six to twelve weeks of training with two to three sessions per week [26]. However, a key question is how these interventions should be designed and tailored to meet the needs of older adults and their ability to engage in PA, and to support behaviour change in the long term. Few data are available on adherence to PA programs among older persons and on tailoring such programs to improve long-term adherence. Personal characteristics, such as age, gender, cultural background, norms, beliefs and health literacy need to be taken into account when designing PA interventions for older adults. Further, social-cognitive variables, in particular self-efficacy, outcome expectations and social support are important for PA initiation and maintenance [27]. In addition, the fear of falling can hamper PA while on the other hand exercises can reduce an individual’s fear of falling [7]. Gender is an important stratifying category in terms of needs, access, targeting and uptake of preventive interventions and should be considered during planning, in the process steps as well as in the evaluation of interventions [28].

#### 1.1.2. Fostering Community Readiness and Participation

Community factors such as capacity building and community involvement are often recommended as key factors to improve equitable access to prevention and health promotion. A systematic review by Stith et al. concluded that four conditions are essential for the successful implementation of a preventive intervention in a community: (i) there is sufficient community capacity, i.e., a functioning community coalition is in place; (ii) the community recognizes a problem and existing programs cannot solve it sufficiently; (iii) a key person/organization is identified; and (iv) there is an appropriate climate for the implementation, i.e., stakeholders will benefit from participation or there will at least be no disadvantages for them, and participation will not be deemed to be too costly [29]. It is therefore recommended to assess and, if necessary, increase community readiness before starting an intervention [29]. Participatory models of intervention development, such as the PRECEDE-PROCEED planning model [30], have been proposed for setting up health promotion programmes that fit to local contexts.

#### 1.1.3. Strengthening Intersectoral Collaboration

The built environment has a crucial impact on PA in terms of accessibility to green spaces and the option to use public urban space, as well as the provision of social infrastructure and local supply [31]. A community environment that favours PA may even produce additional health benefits such as good mental health and injury prevention [32]. In older adults, the neighbourhood environment influences functional health and quality of life. Older people are more likely to be physically active outside in neighbourhoods with high walkability, interconnected street layouts, smooth footways, local services and facilities, and green spaces [33].

The built environment includes urban design, transportation systems and land-use planning. The promotion of PA by urban planners may occur through the consideration of population density, diversity of land use and street connectivity. Though there is multifaceted evidence on the effectiveness of urban environment interventions on PA, this evidence is limited as most studies were conducted in dense, urban environments [34]. Whether the results are also applicable to small towns and cities in more rural areas remains to be determined.

In urban planning, Strategic Environmental Assessment (SEA) at the strategy, policy and programme level, and Environmental Impact Assessment (EIA) at the project level have traditionally addressed health issues. However, SEA and EIA were only two of many aspects and often with a restricted focus on bio-physical health determinants [35]. Though urban health issues are tacitly agreed upon in local planning concepts, they are not yet part of public participation procedures, nor are they implemented explicitly [34]. Moreover, concrete and manageable health impact assessment (HIA) scales, particularly in the environmental assessment, are lacking.

Along with planning aspects, factors such as housing conditions, self-perceived social support, social activity and the capability to organise one’s own daily life have a crucial effect on health behaviour and PA [36]. The work of Sallis and colleagues shows that in the age group of 65 and above, the living environment and housing conditions increasingly gain importance [37]. Therefore, these factors should be considered when designing age-specific interventions to promote PA in older age.

#### 1.1.4. Use of New Technological Devices

Over the last years new technological means (e.g., smart phones, tablet computers) have offered possibilities to increase opportunities for delivering and tailoring interventions. Participation in such technology-supported interventions is associated with a more active lifestyle among young adults [38]. Whether this also applies to older adults is still unclear. The European Commission recently prioritised research in this field, particularly in the area of ‘healthy ageing’. One priority is research on the development of technology-based interventions for health promotion for older European adults [39].

Using the internet and smart phone applications (apps) for health promotion and for primary prevention in healthy populations are promising options for public health researchers because so called ‘small changes’ or ‘early nudges’ approaches can be easily implemented using these modalities, and large amounts of data on health behaviour can be collected [40].

Further advantages include the notion that such intervention modules are accessible and usable independent of an intervention team. Further, such application can reach segments of the population that may not get to know about traditional health promotion interventions [40]. Recent research shows that technologically-supported interventions to promote PA, e.g., using smartphones and mobile devices, may lead to increases in PA among older adults [41].

For many years technology has been used for personal health, e.g., to improve doctor-patient-relationships, for the management of chronic diseases or to support health behaviour change interventions. Smart health systems such as activity trackers, sports watches or networked scales became available only recently [42]. These systems allow the monitoring of relevant health parameters and deliver additional services through the internet. By design these are lifestyle products suitable for daily use by laypersons. Smart health systems, in conjunction with information technologies such as computers and smartphones, hold considerable potential for primary prevention [43].

Applying technology for personal health always requires an interdisciplinary approach, taking into account medical, technological and psychosocial perspectives [44]. Primary prevention places particular emphasis on these perspectives. Usability, acceptance and integration in daily life are crucial to ensure long-term compliance, and may require trade-offs on the medical effects.

Technology can support PA interventions in various ways. Ambient or body-worn sensors allow for the monitoring and objectively quantification of activities [45]. The collected data form the basis for the assessment of the health status and for decisions about future activities. Whereas motivating and persuasive measures, social networking and gamification elements can improve users’ adherence to interventions, background information on, e.g., activity levels may help users understand and master their personal health.

In order to realise the full potential of technology-based health services, users’ acceptance has to be reviewed continuously as one important reason for the failure of technological products supporting individual health is a lack of understanding users’ requirements [46]. Web-based or smart phone based interventions to promote PA might be more useful for ‘younger’ older adults (<80 years of age) who tend to be more experienced in interacting with technology compared to the very old.

#### 1.1.5. Intervention Generated Inequalities

There is a growing body of evidence suggesting that PA levels may be lower among socially disadvantaged population groups compared to more advantaged ones [47]. In this context, social disadvantage can relate to socio-economic (e.g., education/health literacy, income, occupation), socio-cultural (i.e., gender, ethnicity, religion) as well as socio-geographical aspects (e.g., neighbourhood deprivation, social capital). For example, previous research focusing on older adults has shown associations between low physical activity and low education, low wealth, female sex, not being white, and living in a deprived residential area [48,49,50,51,52]. Therefore, social inequalities in health and their determinants, such as PA, are among the main challenges in public health and health promotion, and require consideration in intervention designs.

Health promotion and prevention measures aiming to increase PA can be designed to specifically target socially disadvantaged groups. However, such targeted interventions have been shown to be difficult to implement. On the other hand, there is also evidence suggesting that non-targeted interventions, even if they are successful at improving health or health behaviours overall, may widen social inequalities by benefitting socially disadvantaged groups less [53,54]. These effects have been termed ‘intervention-generated inequalities’ (IGIs) and may arise at several points of intervention implementation, such as service provision, access, uptake, compliance or intervention efficacy [55]. For PA interventions, the issue of IGIs has been discussed with regard to unequal uptake and participation. However, to date only a few studies have examined whether the effectiveness of PA interventions differs between social groups [56,57]. Furthermore, the need for a greater exploration of equity impacts of interventions to promote physical activity among older adults was highlighted by a recent systematic review on interventions for adults around retirement age [58].

A recently published Cochrane review on the effectiveness of community-wide interventions aiming to increase PA underlined this knowledge gap [59]. The authors reported that none of the included studies provided results on socio-economic inequalities or other indicators of health equity thus making it impossible to analyse equity impacts.

Consequently, there is a need to design and implement interventions that also take socially disadvantaged groups into account as well as to systematically include an equity impact assessment in the evaluation of such interventions.

## 2. Materials and Methods

AEQUIPA comprises six universities and two research institutes, forming a prevention research network in North-West Germany. It combines rural and urban areas and two federal states, and merges expertise from social epidemiology, public health, psychology, urban planning, sports sciences, information technologies, health economics and geriatrics.

Social-ecological models provide a comprehensive perspective on analysing PA behaviour and are often recommended as an approach for PA interventions [24,60]. Drawing on this comprehensive perspective, AEQUIPA assesses factors related to PA on different ecological level, ranging from the intra-personal level (e.g., stages of change, technical affinity) over the perceived environment and the characteristics of the behavioural setting (e.g., community readiness) to the policy environment (urban planning). In the same way, the interventions that are developed in the AEQUIPA subprojects address the different ecological levels with some interventions focussing more on the individual level and some more on the contextual level (Table 1). Nevertheless, the comprehensive socio-ecological perspective is maintained by linking interventions addressing different levels (subproject Ready to Change (RTC) and PROMOTE) of developing a multilevel intervention in a single subproject (OUTDOOR ACTIVE). Extensive data collection will enable us to assess how factors on different ecological levels influence PA among older adults.

### 2.1. Research Design

**Figure 1:**
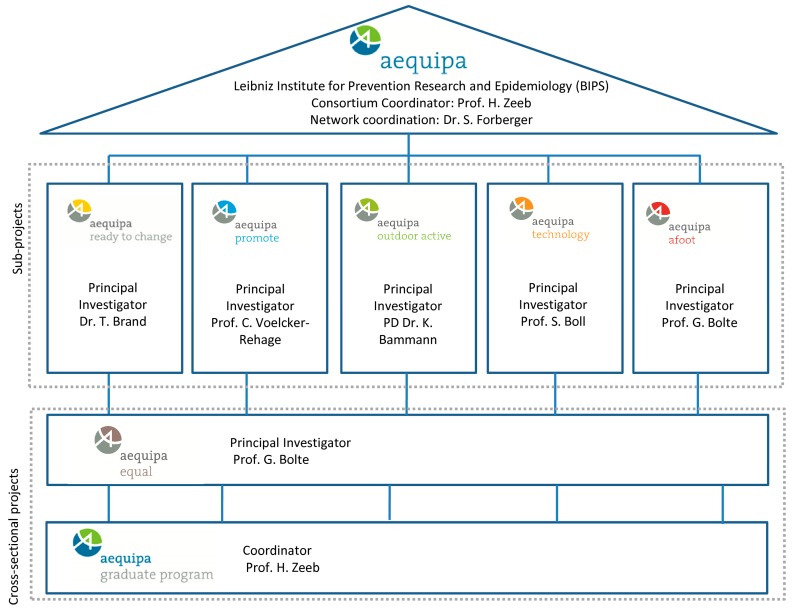
AEQUIPA 1 network structure.

To approach the key challenges, AEQUIPA covers five subprojects, one crosscutting project, and one PhD program (Figure 1). Every subproject is dedicated to its own particular research question and main focus (Table 1). 

However, the internal structure and network approach allow a joint and mutually supportive work on the overarching research question on how to promote and maintain PA in older adults. Overall, the scientific aims of the AEQUIPA network are:

**-**  To strengthen the evidence base for preventive physical activity in the context of healthy ageing in Germany, including health economic aspects (subprojects RTC, PROMOTE).
**-**  To conduct research regarding the environmental, contextual and individual conditions enabling physical activity interventions among older adults (subprojects RTC, PROMOTE, OUTDOOR ACTIVE).
**-**  To implement a strategic linkage of urban planning and public health strategies at the local level to facilitate physical activity of older adults in the context of the built environment (subproject AFOOT).
**-**  To develop new approaches for understanding and monitoring the impact of physical activity interventions on health equity (subproject EQUAL).
**-**  To investigate the role of new technologies in supporting physical activity among older adults (subprojects TECHNOLOGY, PROMOTE).

Where possible, a participatory approach is used within the subprojects. For example, PROMOTE uses target group involvement for the development of its preventive PA program and OUTDOOR ACTIVE applies the PRECEDE-PROCEED model to develop a community-based PA promotion program. Within TECHNOLOGY close cooperation and technic tests are used with and by the participants to analyse technic acceptance and usability.

### 2.2. Measures

AEQUIPA uses various methodological approaches und constructs/instruments (Table 1 and Table 2). Within the empirical studies, persons aged 65 years and above from the German general population and who are able to engage in PA are included in the study. Persons not living independently and who are unable to engage in PA or have severe diseases such as heart attack, stroke and severe asthma are excluded. In general, a physician should be consulted beforehand. For the studies using contextual approaches persons/settings for persons older than 65 years are included/analysed. Detailed inclusion and exclusion criteria are defined by the respective subprojects [61,62]. Constructs and instruments used within the subprojects were kept compatible between the subprojects as far as possible. The methods used are questionnaires, behavioural measurements and trackers, cognitive and physical functioning tests, interviews, focus groups and simulation games. For the data collection randomised controlled trials, intervention and observational studies are being performed along with expert interviews, round tables, workshops and simulation games.

#### 2.2.1. Individual Characteristics

Demographic characteristics are assessed via questionnaires covering age, gender, education, marital status and income. Socio-demographic context variables are assessed in accordance to the DEGS study. Questions regarding education are in line with the International Standard Classification of Education (ISCED).

Health risks and resources are covered by three subprojects and include potentially modifiable behavioural risk factors (e.g., dietary habits, tobacco use, alcohol consumption). Various aspects of mental health and subjective health are collected via self-administered questionnaires based on standardised instruments (Table 2).

Objective measures like weight and height are taken directly by the interviewer. Body composition (bio-impedance analysis), blood pressure (measured with an Omron 705 II (Omron, Mannheim, Germany) and waist circumference are assessed.

Physical fitness assessments comprise aerobic fitness tests, grip strength, reactivity, agility, balance and postural control and flexibility tests. In several subprojects, both self-reported and objectively measured PA data are collected (Table 2).

Variables covering technological aspects are measured in three subprojects (PROMOTE, OUTDOOR ACTIVE, TECHNOLOGY). Technological factors comprise computer experience, usage and usability of, as well as satisfaction with apps, websites and technology in general. Where feasible, the applicability of the devices and their performance for diagnostic and prevention purposes are assessed. Within the intervention, assistance by technical devices, apps and websites as well as usability and satisfaction are assessed via self-developed questionnaires. Because these variables are influenced by multiple factors such as openness, user acceptance and subjective rating, they are classified under individual characteristics. This appears appropriate because the potential for being shaped by social influences and trends is moderated by personal traits.

#### 2.2.2. Contextual Factors

All subprojects collect data on contextual factors such as environmental factors, community situation and living and social conditions from different perspectives. For example, while RTC and AFOOT [61,62] analyse these variables under the focus of community regulations and local government, PROMOTE and OUTDOOR ACTIVE focus on the role of environmental factors (walkability) for PA promotion and maintenance. Functional capacities relevant to daily life activities such as the need for social support, ability to have social activities and relationships, as well as factors such as housing conditions and environment, place attachment and neighbourhood are assessed by standardised self-administered questionnaires (Table 2).

#### 2.2.3. Inequality

Socioeconomic characteristics are assessed at individual and community level. Data on gender, immigration background, education, income and employment are collected within the sub-projects. Inequality may also play a role in the analysis of urban and transport planning regulations and applications, and is assessed through interviews with experts and stakeholders in the respective subprojects.

## 3. Preliminary Results

The AEQUIPA project started in 2015. The final results will be available in 2018. So far, the network has achieved a strong regional linkage with communities, local stakeholders and individuals.

Broad assessments of community readiness for change towards improved PA of the elderly have been conducted (RTC subproject) and the information was used to prepare and implement the PROMOTE trial. In the trial, IT-based approaches to support PA behavior change in the target population were investigated. Within RTC, 118 semi-structured interviews with key informants from 23 municipalities, such as persons from public authorities, community centers, elderly advisory boards, or sports clubs, were conducted. Among communities with low Community Readiness score, local stakeholders were contacted and working groups were installed. The willingness and preparedness to engage among the intervention municipalities differed. For example, in one community an existing working group incorporated the topic of PA promotion into their permanent agenda. In another community, stakeholders decided not to form a working group after a first meeting.

In PROMOTE the effects of an IT-based intervention for PA promotion compared to a waiting list control group was examined. So far 168 persons participated in the first wave of data collection that occurred between May and September 2016. The median age of the participants was 70 years. 55.7% of the participants were female. A second wave started in January 2017 and will end in October 2017. Analyses of intervention effectiveness are planned for the end of 2017.

In the OUTDOOR ACTIVE trial 916 participants were included in the initial survey and the subsequent theory-based development of appropriate community-based interventions. The median age of the participants was 70 years. 51.1% of the participants were female. The data gives insight into the interplay between individual factors, perceived and objective environmental factors and PA. In 2017, the data will be enriched by follow-up data, and thus become a valuable resource for further analyses. Qualitative and quantitative data are being analyzed in the subproject to identify risk profiles for low PA, and also to gain information on how to approach this age group for PA intervention (e.g., barriers and drivers, communication channels, psychological and social profiles).

Individual behavior is the main focus of the TECHNOLOGY subproject which investigates very early preventive interventions to act on pre-frailty, including new IT technologies. 250 participants were included during the first two assessments in 2016. The median age of the participants was 75 years. 59.0% of the participants were female. A third assessment will take place between July and December 2017 and data analyses will follow.

In the AFOOT study extensive work was done to understand planning processes and entry points for health and equity assessment in selected communities so that administrative processes and structures that support primary prevention are strengthened and adapted.

Realizing the potential of all primary prevention interventions to affect equity in health, training and design support focusing on equitable ways of participant recruitment as well as on assessment of social status among participants are offered across all projects. This was done under the guidance of the EQUAL subproject and aimed to offer an empirical basis for health equity assessment. Findings of an EQUAL lead literature review suggest that many studies on universal interventions to promote PA among older adults have not exploited the potential for assessing equity impacts [88].

## 4. Discussion

PA is an important contributor to healthy ageing. Increasing the proportion of healthy and active, independently living older people is not only a way to reduce the pressure on social and welfare systems, but also an overall goal of modern liveable societies. To achieve these goals, evidence-based age-specific PA interventions that, in addition to individual characteristics, also take contextual and technological factors as well as the impact of intervention on inequality into account, are needed. The work of the AEQUIPA network is expected to provide new knowledge on effective primary prevention through the better understanding of mechanisms and improved promotion of PA in the continuously growing group of older adults. It will also provide new insights into the targeting and tailoring of interventions and will extensively test the application of participatory models of intervention development and implementation. AEQUIPA thus addresses central paradigms of prevention and health promotion and explicitly uses theory guidance to develop and implement PA interventions. The aim is to better understand, and thus be able to act upon, social stratification in access to and uptake of PA interventions by an ageing population.

Our research will also investigate the potential role of innovations such as new communication and measurement technologies. New technological developments e.g., activity trackers or sport watches, might foster PA. The potential role of the said innovations will be analysed for its usability in prevention of inactivity and physical degeneration within AEQUIPA. Interventions that are based on or supported by technology will be developed, tailored and tested to strengthen the critical evidence base on new programs planning to incorporate such tools to support PA. Based on the evidence generated through AEQUIPA, we hope to better understand if and how the use of technological innovations among older adults differs from younger persons. For example, focus on benefits rather than costs have been reported for elderly [89]. In addition, the difference in use of technology between men and women is of interest should technology be used as a prevention tool. Further, the role, design and usability of assistive technology can be taken into account and evaluated in the interventional and user context [90]. However, beyond these aspects of technological innovations in PA related preventions among older adults, users’ behaviour plays an important role for all interventions and offers supported by technological products [91].

As a further innovative feature, an integrated approach of public health and urban planning is included in the network. This cooperation will help to identify and improve characteristics of the built environment relevant to PA. This approach considers the perspectives of local stakeholders of spatial planning and administrative action to enable sustainable implementation within the region. Further, AEQUIPA allows a comparison of urban and rural regions in two federal states. A guide for intersectoral policy actions will be developed as intervention measure taking the institutional and administrative framework of urban planning and public health as well as limited communal financial resources into account [61].

AEQUIPA researchers are aware that the question of “fun and enjoy” of PA interventions is important for the sustainability of PA, although it is still under-researched. This aspect is not well captured in AEQUIPA as well and should play a greater role in further studies. We are also aware that the wealth of instruments applied in the different studies is extensive and may work against high-spirited voluntary participation and act more as deterrent. Furthermore, the role of PA occurring daily and in less concrete and focused manners is not well captured in the AEQUIPA research program. While within PROMOTE an exercise program was developed, exercises which can be incorporated in the daily routine are not involved; e.g., storing coffee on the upmost kitchen shelf while having the corresponding filters on the lowest shelf to train balance, muscle strength, and balance during ones daily routine. While inequality effects of interventions are studied within the network, the developed concepts and methodological approaches need to be tested and evaluated further and in other contexts.

The results of the intervention evaluation will add to the discourse on intervention design, particularly on how to tailor interventions to the specific needs of older people. However, disabled people and special groups are underrepresented in our work and hence should be considered in future studies.

The ambitious quantitative design of the network and the multifaceted intervention design could lead to a low response rate with a subsequent adverse impact on the evaluability of effects. It is possible that the planned sample sizes within the subprojects will not be reached. The recruitment covers people older than 65 years and particularly focuses on minorities and marginalized groups (low socioeconomic status (SES)—low level of education and occupational prestige), persons with migrant background, no/low PA level which can often not be easily reached; e.g., not responding to participation invitation particularly when written, are not active and generally not interested, have a different cultural understanding. Several measures were taken to facilitate participant recruitment and retention. Community stakeholders and local minority group organizations were approached during the recruitment process. However, the participation among certain groups, e.g., migrants remained low. To stay in touch with the included participants, public events were organized where the progress of the studies were presented (TECHNOLOGY, OUTDOOR ACTIVE). In the PROMOTE intervention study, regular group sessions are provided to increase participant retention. Furthermore, a hotline was installed to provide assistance to study participants in case of technical problems. Nevertheless, our first experiences call for a systematic reflection of these problems. As a consequence, more intensive community participation and a further tailoring of the developed interventions are planned for the next phase of this project. Additionally, despite the fact that it has a sound theoretical foundation and evidence-base, the intervention design might be too complex.

## 5. Conclusions

All in all, the research results foreseen to be generated in this network will directly address national and international health targets for healthy ageing. The research outcomes are also of key relevance to the strengthening of the prevention in the health care system as they will contribute to an evidence-based development and selection of intervention strategies for PA for older adults. The AEQUIPA project started in 2015 and has now completed its second year. The final results will be available in 2018.

## Figures and Tables

**Table 1:** Physical Activity and Health Equity (AEQUIPA) subprojects, research aim, design/methods used, envisaged sample size and main factors examined.

Subproject Title	Research Aim	Design/Methods Used	Sample Size (Envisaged)	Main Factors Examined
(1) RTC	- To assess the community readiness for promoting PA among older adults - To investigate the efficacy and cost-effectiveness of strategies to increase community readiness to engage older adults in PA interventions - To examine reasons for non-participation in existing PA interventions	Community readiness assessment, expert interviews	Interviews in *n* = 23 municipalities (rural *n* = 12; urban *n* = 11) with 5–6 representatives per municipality	Contextual
(2) PROMOTE	- To assess the perceived community readiness for promoting PA among older adults - To investigate the efficacy and cost-effectiveness of strategies to increase community readiness to engage older adults in PA interventions - To examine reasons for non-participation in existing PA interventions	Randomised controlled trial	Older adults aged 65–75 (*n* = 1000); urban community	Individual, contextual
(3) OUTDOOR ACTIVE	- To assess prevalence and associations of individual and environmental factors regarding outdoor PA in older adults - To identify factors predisposing, enabling and reinforcing outdoor PA in older adults - To develop, implement and evaluate a program for promoting outdoor PA	PRECEDE-PROCEED model, complete survey in reference community, round tables	Complete survey of reference community adults aged 65–75 (*n* = 1000); urban community	Individual, contextual
(4) TECHNOLOGY	- To investigate how IT-based health technologies can be used for primary prevention - To develop a combination of sensors to support preventive measures - To further develop a sensor-based screening tool for functional decline - To adapt and customize the technologies to different users’ socioeconomic background	Observational and intervention study	Observational study older adults aged 65+ (*n* = 250) Experimental intervention study; urban community	Individual, contextual
(5) AFOOT	- To identify local urban strategies and indicators for walkability for health and equity assessment in urban planning procedures - To develop a guideline for intersectoral policy actions taking the institutional and administrative framework of urban planning and public health as well as limited communal financial resources into account	Expert interviews, simulation game, round table, workshops	Expert interviews (*n* = 20) Workshops (*n* = 3) with up to 12 participants Simulation game (*n* = 2–3); urban and rural communities	Contextual
(6) EQUAL	- To review the equity effects of PA interventions among older adults - To provide guidance for the AEQUIPA subprojects on inequalities-sensitive planning and implementation of interventions - To develop methods for an equity impact assessments of the AEQUIPA interventions	Expert interviews, systematic literature review, methods synthesis	Expert interviews (*n* = 5–10)	Individual, contextual

RTC: Ready to Change; PA: physical activity.

**Table 2:** Factors, constructs and instruments/methods used within AEQUIPA sub-projects ^a^.

Factor	Construct	Instrument	Data Collection Mode
**Individual**	**Health**		
	Body weight and height	Stadiometer Seca 217 (Seca, Hamburg, Germany)	M/T
	Obesity, body composition	Waist circumference (Seca 200 (Seca, Hamburg, Germany)); Mid upper arm circumference (Seca 200); Skin fold triceps (Harpenden Skinfold Calliper (Baty International, West Sussex, UK)); Body composition/muscle mass (bio-impedance analysis)	M/T
	Blood pressure	Omron 705CP II	M/T
	Physical fitness	2-Minute-Step; Chair stand; Contralateral concurrent matching task; 30-s Arm Curl Test; Six-Minute Walk Test; Stair-Climb-Power-Test; Counter Movement Jump; Hand grip; Stick fall test; Timed up and Go; Elderly Fall Screening Test (EFST) [63]; De Morton Mobility Index (DEMMI) [64]; Four test balance scale [65]; Keeping balance while walking backwards on a 3/4.5/6 cm broad beam; Standing as still as possible for 60 s; Chair Sit and Reach; Back Scratch	M/T
	Activities of daily living	Instrumental activities of daily living (iADL) [66]	SFQ
	Quality of life	Satisfaction With Life Scale (SWLS) [67]; Short Form-36 Health Survey (SF-36) [68]	SFQ
	**Physical Activity**		
	Subjective physical activity	International Physical Activity Questionnaire (IPAQ) [69]	SFQ
	Objective physical activity	Accelerometer (ActiGraph (ActiGraph, Pensacola, FL, USA)); Fitbit© (Fitbit Inc. San Francisco, CA, USA)	M/T
	**Health-related behaviour**		
	Dietary habits (fruit/vegetable intake)	Self-developed questionnaire	SFQ
	Smoking	Adapted from German Health Interview and Examination Survey for Adults (DEGS)	SFQ
	Alcohol consumption	Alcohol Use Disorders Identification Test (AUDIT-C) [70]	SFQ
	**Mental health and personality**		
	Personality	Big Five (NEO-FFI), adapted from the European Social Survey 2.1	SFQ
	Depression	Center for Epidemiological Studies Depression Scale (CES-D) [71]	SFQ
	Cognitive tests	Auditory Verbal Learning Test (AVLT) [72]; Flanker test [73]; Random number generation task	M/T
	**Self-perception, intention, planning**		
	Self-description	Physical Self-Description Questionnaire (PSDQ) [74]	SFQ
	Subjective need and demand	Adapted from German Ageing Survey (DEAS)	SFQ
	Fear of falls	Geriatric Fear of Falling Measure (GFFM) [75]	SFQ
	Health behaviour	Self-developed questionnaire	SFQ
	Self-efficacy expectation	Self-developed questionnaire adapted from the Health Action Process (HAPA) model	SFQ
	Risk perception	Adapted from Berlin Risk Appraisal and Health Motivation Study (BRAHMS)	SFQ
	Intention	Self-developed questionnaire adapted from HAPA model	SFQ
	Outcome expectation	Self-developed questionnaire adapted from HAPA model	SFQ
	Planning behaviour	Self-developed questionnaire adapted from HAPA model	SFQ
	**Technology**		
	Computer experience	Self-developed questionnaire	SFQ
	Use of application software (App)	Self-developed questionnaire	SFQ
	Short scale technique	Self-developed questionnaire	SFQ
	Technology usage	Self-developed questionnaire	SFQ
	Satisfaction with usability	Self-developed questionnaire	SFQ
	**Socio-demographics**		
	Age, marital status, household size, household structure	Adapted from DEGS, German National Cohort Study [76]	SFQ
	Immigration background (state of origin, year of immigration, language proficiency)	Adapted from DEGS, German National Cohort Study	SFQ
	Education, income, occupation, employment, retirement	Adapted from DEGS, German National Cohort Study, Survey of Health, Ageing and Retirement in Europe (SHARE)	SFQ
**Contextual**	**Environment**		
	Walkability	IPAQ environmental module, Neighbourhood environment walkability scale (NEWS) [77]	SFQ
	Place attachment	Place Attachment Inventory 1.3 [78]	SFQ
	**Community**		
	Community readiness	Community readiness assessment [79]	Personal interview
	Urban planning and PA	Self-developed instruments	Expert interviews, focus groups, workshop, simulation games
	Health authority and PA	Self-developed instruments	Expert interviews, focus groups, workshop, simulation games
	**Living and social conditions**		
	Housing condition and environment	Adapted from DEGS	SFQ
	Perceived social support	Oslo 3-Items Social Support Scale (OSSS) [80]	SFQ
	Social relationships	Social Network Index [81]	SFQ

AUDIT-C: Alcohol Use Disorders Identification Test; AVLT: Auditory Verbal Learning Test; BRAHMS: Berlin Risk Appraisal and Health Motivation Study [82]; CES-D: Center for Epidemiological Studies Depression Scale; DEAS: German Ageing Survey [83]; DEGS: German Health Interview and Examination Survey for Adults [84]; DEMMI: De Morton Mobility Index; EFST: Elderly Fall Screening Test; ESS: European Social Survey [85]; GFFM: Geriatric Fear of Falling Measure; HAPA: Health Action Process model [86]; iADL: Instrumental activities of daily living; IPAQ: International Physical Activity Questionnaire; M/T: measurements and tests; NEWS: Neighbourhood environment walkability scale; OSSS: Oslo 3-Items Social Support Scale; PSDQ: Physical Self-Description. Questionnaire; SF-36: Short Form-36 Health Survey; SFQ: self-administered questionnaire; SHARE: Survey of Health, Ageing and Retirement in Europe [87]; SWLS: Satisfaction with Life Scale; ^a^: The instruments/methods used can vary slightly between the subprojects.
